# Self-reported adherence to foot care in type 2 diabetes patients: do illness representations and distress matter?

**DOI:** 10.1017/S1463423618000531

**Published:** 2018-08-10

**Authors:** M. Graça Pereira, Susana Pedras, Gabriela Ferreira

**Affiliations:** School of Psychology, University of Minho, Braga, Portugal.

**Keywords:** anxiety, depression, illness representations, self-reported foot care adherence, type 2 diabetes

## Abstract

**Aim:**

This study examined the differences and the predictive role of clinical variables, illness representations, anxiety, and depression symptoms, on self-reported foot care adherence, in patients recently diagnosed with type 2 diabetes mellitus (T2DM) and assessed no longer than a year after the diagnosis (T1) and four months later (T2).

**Background:**

The high rate of diabetes worldwide is one of the major public health challenges. Foot care is the behavior least performed by patients although regular foot care could prevent complications such as diabetic foot and amputation. Psychosocial processes such as illness representations and distress symptoms may contribute to explain adherence to foot self-care behaviors.

**Methods:**

This is a longitudinal study with two assessment moments. The sample included 271 patients, who answered the Revised Summary of Diabetes Self-Care Activities, Brief-Illness Perception Questionnaire, and Hospital Anxiety and Depression Scale.

**Findings:**

Patients reported better foot care adherence at T2. Having a higher duration of T2DM and the perception of more consequences of diabetes were associated with better self-reported foot care adherence, at T1. At T2, the predictors were lower levels of HbA1c, better self-reported foot care adherence at T1, higher comprehension about T2DM, as well as fewer depressive symptoms. Interventions to promote adherence to foot care should have in consideration these variables. The results of the present study may help health professionals in designing interventions that early detect depressive symptoms and address illness beliefs, in order to promote foot self-care behaviors reducing the incidence of future complications.

## Introduction

The high rate of diabetes worldwide is one of the major public health challenges affecting 422 million people [World Health Organization (WHO), 2016]. Diabetes is the leading cause of blindness, kidney failure, heart attacks, stroke, and lower limb amputations and was responsible for 1.5 million deaths in 2012 (WHO, 2016). Type 2 diabetes mellitus (T2DM) implies healthy lifestyle changes (physical activity, healthy eating), disease self-management (taking and managing medication, self-monitoring of blood glucose), and prevention of diabetes complications (American Diabetes Association, 2018). Non-adherence to foot care, in particular, may result in serious long-term complications, such as diabetic foot ulcer (DFU), that may be classified as low, medium, or high risk according to the presence of neuropathy, peripheral artery disease or foot deformity, previous history of foot ulceration, or amputation (Game *et al*., 2016). However, according to several studies, foot care is the self-care behavior least performed by patients (Dikeukwu and Omole, 2013; Chiwanga and Njelekela, 2015) and the one with most preventable consequences (Bus and van Netten, 2016). Foot care behavior changes seem to be short-lived (Vileikyte *et al*., 2006), probably because interventions are not focused on psychosocial processes underlying patients’ foot self-care behaviors, such as illness representations and anxiety/depression symptoms.

### Illness representations

The illness representation model (Leventhal *et al*., 2003) advocates patients’ active role in managing their own condition. The model provides a theoretical framework for understanding and predicting patients’ self-care activities (Lawson *et al*., 2008; Harvey and Lawson, 2009). A vast literature has documented that illness representations are associated with adherence to diabetes self-care behaviors (Abubakari *et al*., 2011; Broadbent *et al*., 2011) and with overall quality of life, in patients with diabetes (Mumu *et al*., 2014; Haanstra *et al*., 2015). Few studies have explored the association between illness representations and adherence to foot self-care in newly diagnosed patients and they have not found a consensual framework (Vileikyte *et al*., 2006; van Puffelen *et al*., 2015). Notwithstanding, adherence to foot self-care behavior has been associated with more symptoms attributed to diabetes, stronger beliefs about diabetes as a chronic condition with unpredictable symptoms, as well as feelings of being emotionally upset, a poor understanding of diabetes, and stronger beliefs about personal control regarding diabetes (Vedhara *et al*., 2014; van Puffelen *et al*., 2015).

The existing studies have explored the role of illness representations, mainly in patients at risk or with severe diabetic foot lesions (Mc Sharry *et al*., 2011; Van Esch *et al*., 2014; Kugbey *et al*., 2015; van Puffelen *et al*., 2015; Kugbey *et al*., 2017). Also, engagement in foot care has been reported more often in patients with complications (van Puffelen *et al*., 2015). Nevertheless, adherence to foot self-care behaviors, in newly diagnosed patients, is mandatory in order to prevent DFU, given the global DFU prevalence of 6.3% (95% confidence intervals: 5.4–7.3%) (Zhang *et al*., 2017). Also, recently, Vedhara *et al*. (2016) found that illness beliefs had a significant independent effect on survival in patients with diabetes and foot ulceration suggesting that illness beliefs could be the focus for future therapeutic interventions to improve survival and, in our view, to improve adherence to foot care as well.

### Psychological distress: anxiety and depression symptoms

After diabetes diagnosis, anxiety and depression symptoms have been reported by patients and may function as a response to the perceived threat of mandatory lifestyle changes and to the possible long-term disease complications (de Groot *et al*., 2010; Chen *et al*., 2011; Bogner *et al*., 2012; Balhara and Verma, 2013). In fact, in newly diagnosed patients, anxiety and depression symptoms are extremely common (Bajaj *et al*., 2012; Chen *et al*., 2013) since, in addition to the need to adapt to self-care behaviors and consequent life changes, patients also need to adjust psychologically to a chronic disease diagnosis. Besides, anxiety and depression symptoms interfere with adherence to diabetes self-care behaviors (Khuwaja *et al*., 2010; Papelbaum *et al*., 2011) that may lead to decreased health and quality of life (Khuwaja *et al*., 2010).

The literature regarding the association between depression symptoms and foot care non-adherence shows mixed results. In some studies, there was a significant relationship between depressive symptoms and foot examination (Coelho *et al*., 2014; Rivera-Hernandez, 2014) but in others, this result was not found (Hernandez *et al*., 2016; Udovichenko *et al*., 2017). However, studies are consensual when describing the presence of depression in patients with a first DFU (Ismail *et al*., 2007; Gonzalez *et al*., 2010). Also Williams *et al*. (2010) in a prospective study, showed that the risk of having a foot ulcer in patients with no previous history of ulcer was two times greater among depressed patients when compared with patients without depression. Unlike depression, studies on anxiety in patients with DFU are limited. In one study, patients with primary healed ulcers reported less anxiety than those with current ulcers (Ragnarson Tennvall and Apelqvist, 2000) but other studies found no relationship between anxiety and the presence of a DFU (Iversen *et al*., 2009; Udovichenko *et al*., 2017). Diabetic foot and amputation are complications that could be prevented with regular foot self-care behaviors (Bus and van Netten, 2016) but perceptions of a threat or risk may impact the patient’s emotions, which could have an effect on adherence to health care behaviors (van Puffelen *et al*., 2015).

### Metabolic control

At a clinical level, better metabolic control, measured through glycosylated hemoglobin (HbA1c), have been associated with better adherence to self-care behaviors, namely self-monitoring of blood glucose (Schütt *et al*., 2006) and diet (Andrade *et al*., 2017).

### Current study

In this study, self-reported foot care adherence was the dependent variable; the HbA1c level and the duration of diagnosis were clinical independent variables; illness representations, anxiety, and depression symptoms were the psychological independent variables assessed. The present study’s aim was focused on: (i) the differences on self-reported foot care adherence in newly diagnosed patients between the first assessment (T1; up to one year after the diagnosis) and the second assessment (T2; four months later – ie, the next patient’s routine appointment); (ii) and the predictive role of clinical and psychological variables at T1, on self-reported foot care adherence, both at T1 and T2.

Knowing how patients adjust to the disease since its initial stages and what variables predict adherence, as early as possible, will be helpful in designing interventions to promote adherence to T2DM self-care behaviors, preventing future complications.

## Methods

### Sample and procedure

The sample included patients recently diagnosed with T2DM. The study was approved by the Northern Regional Health Care Association in Portugal. All primary care health units were contacted and invited to participate. The data were collected between 2010 and 2013 in 40 health units in the North of Portugal after the approval of the respective Ethic Committees. The health professionals of these units identified the participants eligible for the study. Inclusion criteria comprised having a diagnosis of T2DM no longer than a year before the assessment; being 18 years old or more; taking only oral medication for diabetes, and being ulcer free. The exclusion criteria included having a psychiatric or mental illness recorded in the patient’s clinical chart. Those patients who met the criteria for participation were contacted by the researcher by phone and invited to participate. The inclusion of patients was consecutive. Participation was voluntary and all participants completed an informed consent form and answered the questionnaires the same day of their regular medical appointment.

This study focused on the initial stages of diabetes using a longitudinal design with two assessment moments: T1 that included patients diagnosed in the year previous to the assessment and the T2, four months later – that is, the next routine medical/nursing appointment. Patients in primary health care, in Portugal, receive education about diabetes in medical/nursing appointments once every three months, in their routine consultations based on the guidelines of the International Working Group of Diabetic Foot and described in a guideline of the Portuguese National Health Care System (No: 8/DGCG, 24/04/01). During the period between T1 and T2, patients received only the standard care for diabetes.

### Instruments

Patients answered to the following instruments both at T1 and T2:

#### Revised Summary of Diabetes Self-Care Activities Measure (RSDSCA; Toobert *et al*., 2000)

This scale includes 11 items, assessing levels of self-care and management of various components of diabetes regimen, such as diet (four items), physical activity (two items), glucose monitoring (two items), and foot care (two items). Higher scores indicate better adherence to diabetes self-care behaviors. In the present study, the Portuguese version of the questionnaire (Pereira *et al*., 2008) was administered and only the foot care subscale was included. Self-reported foot care adherence items were: ‘On how many of the last seven days did you check your feet?’ and ‘On how many of the last seven days did you inspect the inside of your shoes?’ with a Likert scale of eight points, ranging between zero (zero days) and seven (seven days). The Cronbach’s *α* in the present study was 0.80.

#### Hospital Anxiety and Depression Scale (HADS; Zigmond and Snaith, 1983)

This scale has 14 items grouped into two subscales that measure anxiety (seven items) and depression (seven items) symptoms. Higher scores indicate more symptoms of depression or anxiety. HADS was validated for Portuguese patients with diabetes (Pais-Ribeiro *et al*., 2007), being a widely used measure in clinical practice in hospital settings that discriminates somatic symptoms from anxiety and depression symptoms. Besides that, HADS is a brief measure, relatively quick and easy to administer, with good psychometric properties. In this sample, Cronbach’s *α* was 0.75 for the depression subscale and 0.77 for the anxiety subscale.

#### Brief-Illness Perception Questionnaire (IPQ-Brief; Broadbent *et al*., 2006)

This scale includes eight items that assess illness representations such as consequences, timeline, personal control, treatment control, identity (symptoms), concerns, comprehension, and emotional response. Higher scores indicate more threatening illness perceptions about T2DM. The Portuguese version of the questionnaire was administered (Figueiras *et al*., 2010). Since each scale only has one item, *α* cannot be computed. Pearson coefficient correlations, as in the original version, were performed. In this sample, the correlations between the subscales were significant ranging between 0.35 and 0.53.

### Data analyses

The frequencies, means and standard deviations of the sample variables were calculated. Pearson correlation coefficients were used to analyze the associations between clinical and psychological variables. In order to analyze the differences in self-reported foot care adherence over time, a *t*-test for paired samples was performed. Effect size was tested through the Cohen’s *d*, where an effect size of 0.20 is considered small, 0.50 moderate, and 0.80 large. To find the associated variables with foot care adherence at T1 and the predictors at T2, two models of hierarchical regression (method enter) were conducted. Since independent variables were highly predictive and related to the dependent variable, procedures to avoid multicollinearity and singularity were adopted, to guarantee that tolerance and Variance Inflation factor values were acceptable (>0.1 and <4, respectively). In the first model, T1 variables were included (duration of diagnosis and HbA1c level in the first step, illness representations about T2DM in the second step, and anxiety and depression in the third step). The second model included T1 variables (self-reported foot care adherence and HbA1c level in the first step, illness representations in the second, and anxiety and depressive symptoms in the third step) predicting the self-reported foot care adherence at T2. The analyses were performed using the IBM SPSS Statistics 24. The level of significance was set at *P*<0.05.

## Results

### Sample characteristics

All patients that met the inclusion criteria were consequently invited and 371 accepted to participate at T1 but only 271 participated at T2. The analysis was performed on the 271 patients that were assessed on both moments. The reasons to drop out from T1 to T2 were due to several reasons: no longer receiving care at the health care unit, missing the medical/nursing appointment, not being able to be contacted. Therefore, the sample consisted of 271 patients diagnosed with T2DM up to 12 months before T1. The socio-demographic and clinical characterization of the sample is presented in Table 1.

**Table 1:** Descriptive statistics for socio-demographic and clinical variables (*n*=271)

Continuous measure	Minimum	Maximum	Mean	SD
Age	33	86	59.16	10.23
Duration of T2DM diagnosis (in months)	1	12	6.22	3.47
HbA1c level (T1)	4.60	14.00	7.00	1.47
HbA1c level (T2)	4.50	13.60	6.73	1.11
Categorical measure				%
Gender				
Female				43.2
Male				56.8
Education level				
Illiterate				5.2
Fourth grade				67.5
Sixth grade				14.4
Ninth grade				9.2
Equal or higher than 12th grade				3.7
Employment status				
Active (employed)				42.6
Inactive (unemployed, retired)				57.5
Marital status				
Married/Living Together				99.3
Duration of T2DM diagnosis				
Till 6 months				57.0
Between 6 and 12 months				43.0

T2DM=type 2 diabetes mellitus.

### Differences in self-reported foot care adherence from T1 to T2

There were significant differences in self-reported foot care adherence between T1 and T2 [*t* (270)=−3.68, *P*<0.001, Cohen’s *d*=0.23]. Patients reported better foot care adherence at T2 (M=7.23, SD=6.08) than at T1 (M=5.89, SD=5.79).

### Relationship among variables

The coefficient correlations between clinical and psychological variables are presented in Table 2. Only the variables significantly associated with foot care adherence at T1 and at T2 were introduced in the respective regression model. Although HbA1c level was not associated with foot care at T1 or T2, the variable was added to both regression models as a predictor of DFU risk.

**Table 2:** Results of Pearson’s coefficient correlation between clinical and psychological variables

Measure	1	2	3	4	5	6	7	8	9	10	11	12	13	14
1. Foot care (T1)	–													
2. Foot care (T2)	0.49***	–												
3. IPQ1 (T1)	0.28***	0.06	–											
4. IPQ2 (T1)	−0.03	0.03	0.14*	–										
5. IPQ3 (T1)	−0.04	−0.16**	−0.01	−0.003	–									
6. IPQ4 (T1)	−0.06	−0.19**	0.22***	−0.16**	0.20***	–								
7. IPQ5 (T1)	0.19***	0.08	0.55***	0.14*	−0.18**	0.06	–							
8. IPQ6 (T1)	0.18**	0.14*	0.41***	0.20***	−0.20***	−0.07	0.31***	–						
9. IPQ7 (T1)	−0.15*	−0.28***	−0.06	−0.24***	0.40***	0.38***	−0.14*	−0.26***	–					
10. IPQ8 (T1)	0.17**	0.10	0.44***	0.21***	0.05	−0.12*	0.29***	0.49***	−0.18**	–				
11. Anxiety (T1)	0.04	−0.002	0.23***	0.11	0.14*	0.01	0.16**	0.16**	−0.04	0.21***	–			
12. Depression (T1)	−0.003	−0.13*	0.18**	0.03	0.19***	0.03	−0.03	−0.04	0.06	0.13*	0.70***	–		
13. HbA1c (T1)	0.01	−0.12	0.17**	0.06	−0.05	−0.02	0.15*	0.05	0.04	0.02	−0.04	−0.10	–	
14. Duration of diagnosis (T1)	0.12*	0.11	−0.006	0.06	−0.03	−0.04	−0.08	−0.03	−0.02	−0.02	−0.01	−0.07	−0.15*	–

IPQ=Illness Perception Questionnaire; IPQ1=consequences; IPQ2=timeline; IPQ3=personal control; IPQ4=treatment control; IPQ5=identity (symptoms); IPQ6=concerns; IPQ7=comprehension; IPQ8=emotional response; HbA1c=glycosylated hemoglobin. *P<0.05; **P⩽0.01; ***P⩽0.001.

### Predictors of self-reported foot care adherence at T1 and T2

To predict the self-reported foot care adherence at T1, the hierarchical regression model included duration of diagnosis and HbA1c level in the first step and the illness representations about T2DM were added in the second step. The final model revealed that duration of T2DM diagnosis and patients’ representations about diabetes’ consequences were predictors of self-reported foot care adherence at T1. Longer duration of T2DM diagnosis and the perception of more consequences of diabetes at T1 were associated to better self-reported foot care adherence, at T1. The model explained 10% of the variance (Table 3).

**Table 3:** Results of hierarchical regressions to predict the self-reported foot care adherence at T1 and at T2

	Self-reported foot care adherence							
	T1				T2			
Predictors	*β*	*t*	*P*	95% CI (lower, upper)	*β*	*t*	*P*	95% CI (lower, upper)
Final model								
HbA1c level (T1)	−0.01	−0.21	0.83	(−0.52, 0.42)	−0.13	−2.49	**0**.**01**	(−0.96, −0.11)
Duration of diagnosis	0.13	2.12	**0**.**04**	(0.01, 0.41)	–	–	–	–
Foot care (T1)	–	–	–	–	0.47	8.92	**<0**.**001**	(0.38, 0.59)
IPQ1 (T1)	0.25	3.23	**0**.**001**	(0.17, 0.69)	–	–	–	–
IPQ3 (T1)	–	–	–	–	−0.04	−0.78	0.44	(−0.32, 0.14)
IPQ4 (T1)	–	–	**–**	–	−0.10	−1.78	0.08	(−0.60, 0.03)
IPQ5 (T1)	0.04	0.50	0.62	(−0.26, 0.44)	–	–	–	–
IPQ6 (T1)	0.03	0.39	0.70	(−0.19, 0.28)	−0.005	−0.09	0.93	(−0.19, 0.17)
IPQ7 (T1)	−0.11	−1.85	0.07	(−0.41, 0.01)	−0.15	−2.42	**0**.**02**	(−0.49, −0.05)
IPQ8 (T1)	0.03	0.48	0.63	(−0.20, 0.32)	–	–	–	–
Depression (T1)	–	–	–	–	−0.12	−2.32	**0**.**02**	(−0.34, −0.03)
*R* ^2^ (adj. *R* ^2^)	0.124 (0.100)				0.323 (0.305)			

HbA1c=glycosylated hemoglobin; foot care (T1)=self-reported foot care adherence at T1; IPQ=Illness Perception Questionnaire; IPQ1=consequences; IPQ3=personal control; IPQ4=treatment control; IPQ5=identity (symptoms); IPQ6=concerns; IPQ7=comprehension; IPQ8=emotional response.

At T2, the regression model included HbA1c level and the self-reported foot care adherence at T1, in the first step, illness representations at T1 in the second step, and depressive symptoms at T1, in the third step. The final model showed that HbA1c level, self-reported foot care adherence, representations about comprehension of T2DM, as well as depressive symptoms were significant predictors. Lower levels of HbA1c, higher levels of adherence to foot care, the perception of higher comprehension about T2DM, as well as lower depressive symptoms, at T1 predicted better self-reported foot care adherence at T2. The model explained 31% of the variance (Table 3).

## Discussion

Results showed that patients reported better foot care adherence at T2 than at T1. Taking into consideration the period of only four months between T1 and T2, this is an interesting finding, which may be explained by the education about self-care behaviors and foot care, provided by the health professionals, that patients received after the diagnosis.

At T1, longer duration of T2DM was associated with better self-reported foot care adherence. A study found decreasing rates of medication adherence in the six months that followed the beginning of treatment, in recently diagnosed patients (Osterberg and Blaschke, 2005). We hypothesize that this result may be explained by the acquired knowledge, provided in the nursing and medical consultations, regarding the importance of foot care adherence to prevent the development of future complications, such as a DFU and amputations. Foot care is a major topic addressed in the routine educational medical/nursing appointments after T2DM diagnosis, by health professionals.

Concerning illness representations, the perception of diabetes consequences was associated with adherence to foot care. However, several studies found that other illness representations, such as T2DM symptoms, the perception of diabetes as a chronic condition and feeling more emotionally upset by the illness, played a role in adherence to foot care (van Puffelen *et al*., 2015). In addition, Abubakari *et al*. (2011) found that the perception of severe T2DM consequences was associated with less foot examination. Thus, the present result may have to do with the sample having been diagnosed no longer than a year, that may have prompt patients to perceive diabetes consequences, paramount as a result of their recent experience with the disease. Notwithstanding, it makes intuitive sense that, if patients perceived T2DM as having serious consequences, they will adhere to foot care to prevent the development of future complications.

Anxiety and depression symptoms were not significantly associated with foot care adherence at T1, which is also an interesting finding. It may be possible that these symptoms of distress are a reaction to the diagnosis of a chronic disease. According to the present results, it appears that illness representations about T2DM consequences have a greater impact on adherence than anxiety and depression, in the initial phase of diagnosis (T1).

Concerning foot care adherence at T2, lower levels of HbA1c and higher levels of self-reported foot care adherence at T1 were the significant predictors. These results are not in accordance with the perspective of perceived treatment efficacy that suggests a tendency for patients not to perform treatment actions, if no visible, tangible, and positive outcomes are apparent (cf. Polonsky and Skinner, 2010). Higher comprehension about T2DM at T1 was also a significant predictor of foot care adherence at T2. However, poor understanding regarding foot ulceration has been associated with better adherence to foot self-care behavior (Vedhara *et al*., 2014). A study also found that diabetes knowledge was a significant predictor of foot care (Kugbey *et al*., 2017). It makes intuitive sense that patients with a greater disease comprehension would adhere more, in order to prevent the development of serious complications, later. Besides, less depressive symptoms predicted foot care adherence at T2, but the literature is not consensual about this relationship. Some studies found a significant negative association between depressive symptoms and foot examination (Coelho *et al*., 2014; Rivera-Hernandez, 2014) while others did not (Hernandez *et al*., 2016; Udovichenko *et al*., 2017). It is known that depressive symptoms may interfere with patients’ cognitive functions (eg, memory) (Danna *et al*., 2016) and, therefore, contribute also to decrease adherence (Khuwaja *et al*., 2010; Papelbaum *et al*., 2011). Therefore, these results highlight the importance of assessing patients’ representations about the consequences and comprehension regarding T2DM, as well as the depressive symptoms, right after the diagnosis, in order to promote adherence to foot care.

The present findings have clinical implications. First, besides providing information and education about foot care and teach patients new skills to promote adherence to foot self-care, health professionals should be aware of the predictive role of illness representations in adherence to foot care, particularly, the perception of more consequences and higher comprehension regarding T2DM. It is also important to assess depressive symptoms in recently diagnosed T2DM patients in order to identify those with a higher risk of non-adherence. However, the ability of most screening measures to identify diabetes-related disorders and depression has been poor (Hermanns *et al*., 2013) and instruments that have an accurate sensitivity for diabetes-specific emotional problems are needed. Also, the published randomized controlled trials revealed valuable efforts to increase patient’s illness perceptions, however, the interventions described were mainly focused on patients with persistently poorly controlled diabetes (Keogh *et al*., 2011; Kugbey *et al*., 2017). According to this study interventions should also be directed to newly diagnosed patients. Interventions such as the DESMOND in newly T2DM patients has been positive for psychosocial outcomes, regarding illness beliefs, although the differences in the biomedical and lifestyle outcomes were only significant at 12 months and not three years later (Khunti *et al*., 2012).

There are some limitations in the present study that need to be addressed. This study only used self-report measures. Also, the IPQ-Brief comprises only one item for each illness representation. This instrument was also adapted regarding general diabetes perceptions and not foot care, in particular. RSDSCA also comprises only two items that assess foot care. However, as far as we know, there is no validated measure to assess adherence to foot care, and most studies have also used this scale. Nevertheless, future studies should use a specific instrument to assess foot care adherence given the specificity of diabetic foot care behaviors (eg, hydrate your feet, but not between your toes; cut your nails gently; wear socks to sleep; do not walk barefoot).

In spite of the involvement of several health care units, the sample size may be considered modest and, therefore, caution regarding the interpretation of results is required. The sample only included T2DM patients on oral medication, married or in a relationship and recently diagnosed (eg, having received two to four medical/nursing appointments at T1 and one more, at T2). Consequently, future research should include patients with longer T2DM duration, taking insulin, single or living alone. Using longitudinal designs over longer periods of time are also required, in order to better understand how illness representations and distress change over time regarding adherence to foot care behaviors, controlling for the number of patients’ consultations and foot risk for DFU.

## Conclusion

This is the first study, as far as the authors are aware, that explores the relationships between illness representations, anxiety and depression symptoms, regarding adherence to foot self-care behaviors, in newly diagnosed patients. According to the present findings, a primary care health professional should assess patients’ perceptions about consequences and comprehension of T2DM, as well as depressive symptoms, since the latter may influence adherence to foot care. Particularly, patients with a perception of lower consequences and comprehension regarding T2DM need to be targeted for intervention. Cognitive-behavioral therapy may be useful in changing or promoting illness beliefs, in order to prevent diabetes complications such as DFUs. Health education programs should also target patients for depressive symptoms. The results of this study emphasize the importance of an early assessment of depression and illness perceptions in the promotion of foot self-care behaviors, in order to reduce future complications.
